# Risk-based scoring and genetic identification for anisakids in frozen fish products from Atlantic FAO areas

**DOI:** 10.1186/s12917-020-02286-7

**Published:** 2020-02-21

**Authors:** Giorgio Smaldone, Elvira Abollo, Raffaele Marrone, Cristian E. M. Bernardi, Claudia Chirollo, Aniello Anastasio, Santiago P. del Hierro

**Affiliations:** 1 Department of Agricultural Sciences, University of Naples, Federico II, via Università 100, 80055 Naples, Portici (NA) Italy; 2 Centro di Riferimento Regionale per la Sicurezza Sanitaria del Pescato CRiSSaP, Naples, Campania Region Italy; 3 Centro Tecnológico del Mar - Fundación CETMAR, C/Eduardo Cabello s/n, 36208 (Pontevedra), Vigo Spain; 4 Department of Veterinary Medicine and Animal Production, Unit of Food Hygiene, University of Naples, Federico II, via Delpino 1, 80137 Naples, Italy; 5 Department of Veterinary Science and Technologies for Food Safety, Laboratory of Food Inspection, Università degli Studi di Milano, Via A. Grasselli, 7-20137, Milano, Italy; 6 Ecobiomar - Instituto de Investigaciones Marinas de Vigo – CSIC - C/ Eduardo Cabello 6, 36208 (Pontevedra),, Vigo Spain

**Keywords:** *Anisakis*, food safety, risk ranking tool, zoonotic risk, fish inspection

## Abstract

**Background:**

The presence of *Anisakis* larvae in fish represents a major public health concern. Effective risk management procedures should be applied to prevent heavily infected products from reaching the market. The aim of the study is to provide preliminary data on parasite exposure and risk classification in frozen fish products by applying a risk categorization scheme (site, abundance, density and epidemiology – SADE) and Fish Parasite Rating (FPR) method. Fish and cephalopods samples (N = 771) from 5 different FAO Atlantic areas were examined and categorized after an accurate visual inspection and a chloro-peptic digestion.

**Results:**

In 25 out of 33 fish species parasite larvae were found. 10897 anisakids larvae were collected and identified to genus level. *Molva dypterygia*, *Conger conger, Zeus faber* and *Aphanopus carbo* were shown to be the most highly infected species. SADE and FPR scores were 1 and poor, respectively, for the referred species, because of the disseminated *Anisakis* infection and commercial rejection.

**Conclusion:**

SADE/FPR method showed high specificity and accuracy. The information provided in this work could be used in early warning systems for the detection of parasites in fishery products and might help fishing industries in establishing management strategies for infected stocks in terms of cost saving decisions.

## Background

Nematodes of the Anisakidae family are fish parasites that can be found all around the world. The larvae live in the gut, visceral peritoneum and flesh of many marine fish and cephalopod species and can colonize through different trophic bridges ensuring and widening the parasite life cycle. Differences in host range, host specificity and pathogenetic potential, even among members of a given sibling species complex, have been historically suggested for anisakids [1–3]. Additionally, a positive relationship between body size/age of fish and larval nematode prevalence and/or abundance has been demonstrated in several commercially important fish species from different wild catch sea areas [4–7].

Because no sea fishing grounds can be considered *Anisakis* free and the infection by anisakid larvae in fish is a natural condition and their complete eradication is not feasible [8], surveillance studies are of great interest to determine the risk exposure for those hot-spot geographic areas of parasite recruitment to fish production value chains. Moreover, nematodes of *Anisakis* genera are zoonotic parasites. In humans the ingestion of Anisakidae larvae can result in infection with live larvae, an allergic reaction to Anisakidae allergens or both [9–12]. The increased consumption of raw or undercooked fish constitutes an underestimated zoonotic potential risk [13–15]. In the last decade, *Anisakis* have been included among the biological hazards reported through the Rapid Alert System for Food and Feed (RASFF) of the European Commission, within the European Union (EU).

European legislation [16] enforces an accurate visual inspection during the official control and in self-monitoring programs to prevent fish borne zoonoses: in this context Food Business Operators (FBO) must ensure that no fishery products obviously contaminated with visible parasites reach the consumers. According to the “Guidance document on the implementation of certain provisions of Regulation (EC) No 853/2004 on the hygiene of food of animal origin” [17], a fishery product is considered obviously contaminated if visible parasites are found in edible portions; however, a maximum number of parasites was not defined. Furthermore, the application of visual inspection procedure in the fishery industry depends on the ability and training of FBO [18]. Because the presence of dead visible parasites could only represent a defect [19, 20] altering the global products quality and in order to comply with the EU prescriptions, in addition to the official control and self-monitoring procedures, the most practical procedure could be the use of a predicting scheme for evaluation of nematode larvae in the edible part of the fish batches as suggested by the European Food Safety Authority (EFSA) [21]. The use of the SADE scheme (acronym of Site of infection; Assurance of quality; Demography - density of parasites; Epidemiology of parasites) proposed by Llarena-reino et al., [22], combined with the Fish Parasite Rating (FPR) method [23], aimed at precisely evaluating the likely outcome of infected fish lots, which could be useful tools. FPR standard is a certified Community Trade Mark - Register No 012266607 at the Office for Harmonization in the Internal Market (OHIM) and provides the staging of fish lots, helping in planning manufacture, commercial, and research decisions during self-management programs. The aim of this research is to provide data on parasite risk exposure in commercial frozen fishery products collected in Atlantic FAO areas using the SADE scheme combined with FPR method in comparison with the official visual inspection procedure.

## Results

### Parasites frequency in fish sub lots

A total of eight fish species (24.24% of sampled fish species/sub lots) were anisakid-free. Nematode larvae were not detected in *Mallotus villosus*, *Glyptocephalus cynoglossus*, *Dicologlossa cuneata*, *Galeoides decadactylus*, *Trachurus trecae*, *Salilota australis*, *Atlantoraja castelnaui* and *Serranus cabrilla*. A total of 10897 anisakid larvae were collected and identified to genus level in the flesh of 25 species. Among these species, *M. dypterygia*, *C. conger*, *Z. faber* and *A. carbo,* coming from FAO area 27 (Northeast Atlantic) were the most highly parasitized (Table 1) showing a total prevalence of infection (P) of 100% with a mean abundance (MA ± SD) of 204.52 ± 91.14, 115.16 ± 96.77, 44.96 ± 32.66, 74.1 ± 28.55 respectively. In these species, 90.45 % of the total larvae were detected: in particular *M. dypterygia* reached the highest density of parasites (102.26 larvae/kg). As much as 46.97 % of total larvae in this species were detected. The statistical analyses indicate that there was a correlation between MA and fish sample mean weight (*p* < 0.001).

**Table 1:** Infection values according to Bush et al. 1997

*Sub lot host species*	*Parasites*		*PREVALENCE (% ± CI 95%)*			*MEAN INTENSITY ± SD*			*MEAN ABUNDANCE ± SD*		
	*Found*	*Correctly classified*	*Epaxial*	*Hypaxial*	*Total*	*Epaxial*	*Hypaxial*	*Total*	*Epaxial*	*Hypaxial*	*Total*
*Alepocephalus bairdii*	14	5	0.00	16.00 ± 0.14	16.00 ± 0.14	0.00	3.50 ± 2.38	3.50 ± 2.38	0.00	0.56 ± 1.55	0.56 ± 1.55
*Aphanopus carbo*	741	6	60.00 ± 0.30	100.00	100.00	1.33 ± 2.56	73.30 ± 28.46	74.10 ± 28.55	0.8 ± 2.29	73.30 ± 28.46	74.1 ± 28.55
*Atlantoraja castelnaui*	0	0	0.00	0.00	0.00	0.00	0.00	0.00	0.00	0.00	0.00
*Caelorinchus fasciatus*	3	3	4.00 ± 0.07	8.00 ± 0.10	12.00 ± 0.12	1.00	1.00	1.00	0.04 ± 0.20	0.08 ±0.27	0.12 ± 0.33
*Clupea harengus*	13	12	0.00	32.00 ± 0.18	32.00 ± 0.18	0.00	1.62 ± 0.74	1.62 ± 0.74	0.00	0.52 ± 0.87	0.52 ± 0.87
*Conger conger*	2879	12	64.00 ± 0.18	100.00	100.00	2.50 ± 2.12	113.56 ± 96.34	115.16 ± 96.77	1.6 ± 2.08	113.56 ± 96.34	115.16 ± 96.77
*Dicologlossa cuneata*	0	0	0.00	0.00	0.00	0.00	0.00	0.00	0.00	0.00	0.00
*Galeoides decadactylus*	0	0	0.00	0.00	0.00	0.00	0.00	0.00	0.00	0.00	0.00
*Genypterus blacoides*	83	11	28.00 ± 0.17	64.00 ± 0.18	80.00 ± 0.15	1.57 ± 0.78	4.50 ± 4.93	4.15 ± 4.76	0.44 ±0.82	2.88 ± 4.48	3.32 ± 4.56
*Glyptocephalus cynoglossus*	0	0	0.00	0.00	0.00	0.00	0.00	0.00	0.00	0.00	0.00
*Illex argentinus*	2	2	10.00 ±0.18			2.00			0.20 ± 0.63		
*Lepidopus caudatus*	44	9	0.00	100.00	100.00	0.00	4.88 ± 3.18	4.88 ± 3.18	0.00	4.88 ± 3.18	4.88 ± 3.18
*Macruronus magellanicus*	3	2	3.57 ± 0.06	3.57 ± 0.06	7.14 ± 0.10	2.00	1.00	1.50 ± 0.70	0.07 ± 0.38	0.03 ± 0.18	0.11 ± 0.41
*Macrurus berglax*	314	13	0.00	84.00 ± 0.14	84.00 ± 0.14	0.00	14.95 ± 16.92	14.95 ± 16.92	0.00	12.56 ± 16.42	12.56 ± 16.42
*Mallotus villosus*	0	0	0.00	0.00	0.00	0.00	0.00	0.00	0.00	0.00	0.00
*Melanogrammus aeglefinus*	7	5	0.00	12.00 ± 0.12	12.00 ± 0.12	0.00	2.33 ± 0.57	2.33 ± 0.57	0.00	0.28 ± 0.79	0.28 ± 0.79
*Merluccius capensis*	6	6	4.00 ± 0.07	16.00 ± 0.14	20.00 ± 0.15	1.00	1.25 ± 0.50	1.20 ± 0.50	0.04 ± 0.20	0.20 ± 0.50	0.24 ± 0.52
*Merluccius hubbsi*	216	14	36.00 ± 0.18	84.00 ± 0.14	84.00 ± 0.14	3.55 ± 6.57	8.66 ± 8.10	10.19 ± 8.79	1.32 ± 4.20	7.28 ± 8.08	8.56 ± 8.90
*Merluccius paradoxus*	15	14	2.94 ± 0.05	23.52 ± 0.14	23.52 ± 0.14	1.00	1.75 ± 0.88	1.875 ± 0.99	0.03 ± 0.17	0.411 ± 0.85	0.44 ± 0.92
*Merluccius polli*	3	3	0.00	10.00 ± 0.10	10.00 ± 0.10	0.00	1.00	1.00	0.00	0.10 ± 0.30	0.10 ± 0.30
*Micromesistius australis*	61	12	11.76 ± 0.15	70.58 ± 0.21	70.58 ± 0.21	1.00	4.91 ± 3.47	5.08 ± 3.34	0.11 ± 0.33	3.47 ± 3.69	3.58 ± 3.65
*Molva dypterygia*	5113	8	36.00	100.00	100.00	2.22 ± 1.48	203.72 ± 90.78	204.52 ± 91.14	0.80 ± 1.38	203.72 ± 90.78	204.52 ± 91.14
*Patagonotothen ramsayi*	7	0	0.00	16.00 ± 0.14	16.00 ± 0.14	0.00	1.75 ±1.50	1.75 ±1.50	0.00	0.28 ± 0.84	0.28 ± 0.84
*Regalecus glesne*	4	3	0.00	36.00 ± 0.28	36.00 ± 0.28	0.00	1.33 ± 0.57	1.33 ± 0.57	0.00	0.36 ± 0.67	0.36 ± 0.67
*Reinharditius hippoglossoides*	125	14	0.00	44.00 ± 0.19	44.00 ± 0.19	0.00	8.92 ± 14.2	8.92 ± 14.2	0.00	5.00 ± 11.39	5.00 ± 11.39
*Salilota australis*	0	0	0.00	0.00	0.00	0.00	0.00	0.00	0.00	0.00	0.00
*Serranus cabrilla*	0	0	0.00	0.00	0.00	0.00	0.00	0.00	0.00	0.00	0.00
*Todarodes angolensis*	3	3	10.00 ±0.18			3.00			0.30 ± 0.94		
*Trachurus trachurus*	68	12	0.00	45.45 ± 0.20	45.45 ± 0.20	0.00	6.80 ± 14.54	6.80 ± 14.54	0.00	3.09 ± 10.13	3.09 ± 10.13
*Trachurus trecae*	0	0	0.00	0.00	0.00	0.00	0.00	0.00	0.00	0.00	0.00
*Trachyrhynchus scabrus*	18	6	0.00	32.00 ± 0.18	32.00 ± 0.18	0.00	2.25 ± 1.83	2.25 ± 1.83	0.00	0.72 ± 1.45	0.72 ± 1.45
*Urophycis Chus*	31	7	10.00 ± 0.10	10.00 ± 0.10	20.00 ± 0.14	1.33 ± 0.57	9.00 ±13.85	5.16 ± 9.72	0.13 ± 0.43	0.90 ± 4.55	1.03 ± 4.52
*Zeus faber*	1124	12	72.00 ± 0.17	100.00	100.00	2.05 ± 1.35	43.48 ± 32.11	44.96 ± 32.66	1.48 ±1.47	43.48 ± 32.11	44.96 ± 32.66

The hypaxial region was the most infected location. In fact, in 11 fish species, anisakid larvae were found only in this region and in general the 98.53% of larvae (n. 10737) were identified in this location. In 50% of the samples, the epaxial infection took place simultaneously with hypaxial location.

### Parasite frequency in fishing areas

P of infection (± CI 95%) MA and mean intensity (MI) (± SD) in the different fishing grounds of the study are reported in Table 2. No parasites were found in FAO area 34. Table 2 shows the comparison of P between different FAO areas: significative statistical differences (*p* < 0.0001) between FAO areas were found. In this study, regarding MA and MI, significative statistical differences between FAO area 27 and the other sampling areas were found (*p* < 0.001).

**Table 2:** FAO areas infection values according to Bush et al. 1997. Comparison of prevalence (*χ*^*2*^ ) of infection between different FAO areas

*FAO areas*	*Individuals*	*Infected*	*P (%)*	*IC (±) 95 %*	*χ*^*2*^	*p*	*MA*	*± SD*	*MI*	*± SD*
Total sampling areas	771	251	32.55	3.30	106.25	*p* < 0.0001	14.13	77.18	43.41	48.46
21	130	41	31.53	7.98			3.61	10.07	11.46	15.33
27	196	111	56.63	6.93			50.57	85.89	89.30	97.91
34	100	0	0.00	0.00			0.00	0.00	0.00	0.00
41	190	63	33.15	6.69			1.97	4.75	5.95	6.68
47	155	36	23.22	6.64			0.89	4.10	3.86	7.89

### Parasite identification

The results show mixed infection in 45.83 % of the fish sub lot examined. All sequences obtained in this study shared 99-100 % nucleotide identity with other sequences of anisakid species deposited in the GenBank (accession ID and web links for each identified parasite, linked to fish species and FAO area, are indicated in the supplementary materials) belonging to *Anisakis simplex sensu stricto*, *Anisakis pegreffii*, *Anisakis typica*, *Anisakis berlandi*, *Pseudoterranova cattani*, *Pseudoterranova decipiens* s.l., *Contracaecum osculatum* s.l. and *Hysterothylacium aduncum*.

In this study *A. simplex* was the main parasite isolated in fishery products from FAO area 21 (100%) and from FAO area 27 (88.40%), while *A. pegreffii* was the main parasite isolated in fishery products from FAO area 41 (65.9%) and from FAO area 47 (63.82%). Fish collected from FAO area 41 showed the highest variability in terms of different species of parasites found.

### Risk categorisation

Table 4 shows inspection data categorized by the SADE and FPR scoring systems. Using visual inspection, 36.36% of the lots didn't meet the EU standards [16] and were rejected. The rejected batches during the naked eye visual inspection present at least 1 visible larva in the edible portion. Over 66 % of fish sub lots have been accepted as they present less than one parasite in the flesh, expressed as MA [24] (Table 1). MA, due to its correlation with P and with the number of samples [25], could be used to estimate the degree of infestation [26], particurarly in the case of fishery products sold in batches. With regard to the naked eye rejected products, 33.33% of the total rejections belong to FAO 27. Not infected fish batches in FAO area 34 were found.

Using the SADE/FPR schemes, 27.27 % of fish sub lots (*M. hubbsi*, *M. australis*, *U. Chus*, *G. blacoides*, *M. paradoxus*, *A. carbo*, *Z. faber*, *C. conger*, *M. dypterygia*) were assigned a low SADE score (from 1 to 3) corresponding to a “poor” FPR standard. Hence, these fish lots must be discarded. The lowest score (SADE 1) was assigned to *A. carbo, Z. faber, C. conger* and *M. dypterygia,* belonging to FAO 27, corresponding to the 44.44 % of the total rejection. No statistical differences (*p* = 0.3711) between SADE/FPR outcomes and visual inspection were observed.

Finally, the non-zoonotic *H. aduncum* (Raphidascaridae) was also detected in *I. argentinus*: this parasite is generally considered not zoonotic, even if a case of invasive gastro-allergic infection was recently reported [27]. This result did not show differences in the application of SADE scoring system because a co-infection with the zoonotic *A. simplex* was noticed.

## Discussion

### Parasites distribution

The high frequency of parasites and MA observed in this study supports that *Anisakis* has the status of component parasite of many fish species and FAO fishing areas. Among the different species of *Anisakis* isolated, *A. simplex* commonly occurred in various ecologically and economically important fish species from Atlantic FAO areas 21, 27 and 41 (Atlantic Northwest, Northeast and Southwest) as reported by Mattiucci et al. [28]. *A. pegreffii* was found in southern Atlantic Ocean (FAO areas 41 and 47) and in FAO area 27 in agreement to previous studies [29–33]. According to Mattiucci et al. [34], *A. typica* can occur from 30° S to 35° N in warmer temperate and tropical waters and this data were confirmed by our findings (FAO area 47). Unlike Mattiucci [30] who highlighted a discontinuous range of distribution of this species including Pacific Canada, Chile, New Zealand waters and the Atlantic South African coast, *A. berlandi* was found only in South-Atlantic (FAO area 41 and FAO area 47). *P. decipiens* s. l., as reported by Szostakowska et al., [35], occurs sporadically and in our work only in 2 fish species were found (*C. conger* and *Z. faber* from FAO area 27) confirming that only parasites belonging to the *P. decipiens* complex are present in the NE Atlantic Ocean*. P. cattani* was found in *G. blacoides* from FAO area 41, in agreement with Timi et al. [36]. *H. aduncum* and *C. osculatum* s.l. were found only in FAO area 41 with low prevalence, in contrast to data reported by Niklitschek et al. [37] in the same sampling area in N= 41 samples of *M. australis*. Furthermore, in the same fish species caught in this area were found only parasites belonging to *Anisakis* genera.

*Anisakis* and *Pseudoterranova* are generally most abundant in European NE Atlantic waters [8]. These are traditionally some of the most productive fishing areas in Europe and the abundance of different hosts at all trophic levels presumably accounts for the overall abundance of the parasites. Differences in infection levels could also be related to the presence of definitive hosts or to host’s feeding habits [1] and to the abundance of obligate intermediate crustacean and/or cephalopods hosts. *M. dypterygia*, *C. Conger* and *Z. faber* were the most highly infected species (rejected after visual inspection and with the lowest SADE/FPR scores), probably because of their relatively high trophic level in FAO area 27 ecosystems, their size (*p* < 0.05) and high quantity of food intake confirm that this fishing area had the strongest effect on larvae infection [9, 38].

Worst results corresponded to this fishing grounds with significative differences in P, MA and MI match this area and the others (*p* < 0.0001). No statistical differences (*p >* 0.05) between FAO areas with low MA and MI (FAO 21, FAO 34, FAO 41 and FAO 47, Table 3) were observed.

**Table 3:** Number and percentage of parasites well sequenced collected in fishery products from different Atlantic areas

	*A. simplex (n./%)*	*A. pegreffii (n./%)*	*P. decipiens s. l. (n./%)*	*H. aduncum (n./%)*	*A. berlandi (n./%)*	*A. typica (n./%)*	*C. osculatum (n./%)*	*P. cattani (n./%)*
FAO 21	34/100%	0%	0%	0%	0%	0%	0%	0%
*Urophycis Chus*	7/100%	0%	0%	0%	0%	0%	0%	0%
*Reinharditius hippoglossoides*	14/100%	0%	0%	0%	0%	0%	0%	0%
*Macrurus berglax*	13/100%	0%	0%	0%	0%	0%	0%	0%
FAO 27	61/88.40%	2/2.89	6/8.69	0%	0%	0%	0%	0%
*Alepocephalus bairdii*	5/100%	0%	0%	0%	0%	0%	0%	0%
*Conger conger*	6/50%	1/8.33%	5/41.66%	0%	0%	0%	0%	0%
*Molva dypterygia*	8/100%	0%	0%	0%	0%	0%	0%	0%
*Zeus faber*	11/91.66%	0%	1/8.33%	0%	0%	0%	0%	0%
*Aphanopus carbo*	6/100%	0%	0%	0%	0%	0%	0%	0%
*Trachyrhynchus scabrus*	6/100%	0%	0%	0%	0%	0%	0%	0%
*Clupea harengus*	12/100%	0%	0%	0%	0%	0%	0%	0%
*Melanogrammus aeglefinus*	5/100%	0%	0%	0%	0%	0%	0%	0%
*Regalecus glesne*	2/66.66%	1/33.33%	0%	0%	0%	0%	0%	0%
FAO 41	1/2.27%	29/65.9%	0%	1/2.27%	8/18.18%	0%	4/9.09%	1/2.27%
*Micromesistius australis*	0%	8/66.66%	0%	0%	3/25%	0	1/8.33%	0%
*Genypterus blacoides*	0%	7/63.63%	0%	0%	3/27.27%	0%	0%	1/9.09%
*Merluccius hubbsi*	0%	10/71.42%	0%	0%	1/7.14%	0%	3/21.42%	0%
*Caelorinchus fasciatus*	0%	2/66.66%	0%	0%	1/33.33%	0%	0%	0%
*Macruronus magellanicus*	0%	2/100%	0%	0%	0%	0%	0%	0%
*Illex argentinus*	1/50%	0%	0%	1/50%	0%	0%	0%	0%
FAO 47	0%	30/63.82%	0%	0%	5/10.63%	12/25.53%	0%	0%
*Trachurus trachurus*	0%	12/100%	0%	0%	0%	0%	0%	0%
*Lepidopus caudatus*	0%	0%	0%	0%	0%	9/100%	0%	0%
*Merluccius paradoxus*	0%	13/92.86%	0%	0%	0%	1/7.14%	0%	0%
*Merluccius capensis*	0%	5/83.33%	0%	0%	1/16.66%	0%	0%	0%
*Todarodes angolensis*	0%	0%	0%	0%	3/100%	0%	0%	0%
*Merluccius polli*	0%	0%	0%	0%	1/33%	2/66.66%	0%	0%

**Table 4:** Inspection data categorized by the SADE and FPR scoring systems

*Host*	*Density* *(n. larvae/kg)*			*Visual inspection outcome*	*SADE code*	*Score*	*FPR score*	*FAO Areas*
	*Epaxial*	*Hypaxial*	*Total*					
*Atlantoraja castelanui*	0	0	0	Accepted	S3A2D2E3	10	Excellent	41
*Dicologlossa cuneata*	0	0	0	Accepted	S3A2D2E3	10	Excellent	34
*Galeoides decadactylus*	0	0	0	Accepted	S3A2D2E3	10	Excellent	34
*Glyptocephalus cynoglossus*	0	0	0	Accepted	S3A2D2E3	10	Excellent	21
*Mallotus villosus*	0	0	0	Accepted	S3A2D2E3	10	Excellent	21
*Salilota australis*	0	0	0	Accepted	S3A2D2E3	10	Excellent	41
*Serranus cabrilla*	0	0	0	Accepted	S3A2D2E3	10	Excellent	47
*Trachurus trecae*	0	0	0	Accepted	S3A2D2E3	10	Excellent	34
*Trachyrhynchus scabrus*	0	4.80	4.80	Accepted	S2A2D1E0	7	Good	27
*Illex argentinus*			0.44	Accepted	S3A2D2E0	7	Good	41
*Todarodes angolensis*			1	Accepted	S3A2D2E0	7	Good	47
*Clupea harengus*	0	1.73	1.73	Accepted	S2A2D2E0	6	Good	27
*Patagonotothen ramsayi*	0	1.4	1.4	Accepted	S2A2D2E0	6	Good	41
*Melanogrammus aeglefinus*	0	0.80	0.80	Accepted	S2A2D2E0	6	Good	27
*Alepocephalus bairdii*	0	0.56	0.56	Accepted	S2A2D2E0	6	Good	27
*Merluccius polli*	0	0.50	0.50	Accepted	S2A2D2E0	6	Good	47
*Regalecus glesne*	0	0.45	0.45	Accepted	S2A2D2E0	6	Good	27
*Lepidopus caudatus*	0	9.70	9.70	Rejected	S2A2D0E0	4	Fair	47
*Trachurus trachurus*	0	7.72	7.72	Rejected	S2A2D0E0	4	Fair	47
*Reinharditius hippoglossoides*	0	7.14	7.14	Rejected	S2A2D0E0	4	Fair	21
*Macrurus berglax*	0	41.86	41.86	Rejected	S2A2D0E0	4	Fair	21
*Merluccius capensis*	0.22	1.14	1.37	Accepted	S0A2D2E0	4	Fair	47
*Caelorinchus fasciatus*	0.13	0.26	0.40	Accepted	S0A2D2E0	4	Fair	41
*Macruronus magellanicus*	0.14	0.07	0.21	Accepted	S0A2D2E0	4	Fair	41
*Merluccius hubbsi*	0.73	4.06	4.80	Rejected	S0A2D1E0	3	Poor	41
*Micromesistius australis*	0.13	4.09	4.23	Rejected	S0A2D1E0	3	Poor	41
*Urophycis Chus*	0.48	3.27	3.75	Rejected	S0A2D1E0	3	Poor	21
*Genypterus blacoides*	0.44	2.88	3.32	Rejected	S0A2D1E0	3	Poor	41
*Merluccius paradoxus*	0.16	2.35	2.52	Accepted	S0A2D1E0	3	Poor	47
*Aphanopus carbo*	1	91.62	92.62	Rejected	S0A1D0E0	1	Poor	27
*Zeus faber*	2.46	72.46	74.93	Rejected	S0A1D0E0	1	Poor	27
*Conger conger*	0.64	45.42	46.06	Rejected	S0A1D0E0	1	Poor	27
*Molva dypterygia*	0.40	101.86	102.26	Rejected	S0A1D0E0	1	Poor	27

**Table 5:** Samples collected from Atlantic FAO areas

*FAO fishing areas*	*Coordinates*	*Host*	*N. boxes / total fish count*	*Individuals sampled (N)*
FAO 21 Atlantic, Northwest		*Glyptocephalus cynoglossus*	1/60	25
FAO 21 Atlantic, Northwest	*48°38'N 45°43'W*	*Macrurus berglax*	2/50	25
FAO 21 Atlantic, Northwest	*46°51'N 47°20'W*	*Mallotus villosus*	1/50	25
FAO 21 Atlantic, Northwest	*48°33'N 45°45'W*	*Reinharditius hippoglossoides*	8/109	25
FAO 21 Atlantic, Northwest	*48°38' N 45°42' W*	*Urophycis chus*	4/114	30
FAO 27 Atlantic, Northeast	*56°13'N 17°34'W*	*Alepocephalus bairdii*	7/52	25
FAO 27 Atlantic, Northeast	*56°13'N 17°35'W*	*Aphanopus carbo*	1/10	10
FAO 27 Atlantic, Northeast	*FAO 27 IIa*	*Clupea harengus*	2/100	25
FAO 27 Atlantic, Northeast	*FAO 27/ VII*	*Conger conger*	6/100	25
FAO 27 Atlantic, Northeast		*Melanogrammus aeglefinus*	1/50	25
FAO 27 Atlantic, Northeast	*54°34'N 17°59'W*	*Molva dypterygia*	11/71	25
FAO 27 Atlantic, Northeast	*58°38'N 15°04'W*	*Regalecus glesne*	2/11	11
FAO 27 Atlantic, Northeast	*FAO 27/XII*	*Trachyrhynchus scabrus*	2/100	25
FAO 27 Atlantic, Northeast		*Zeus faber*	1/50	25
FAO 34 Atlantic, Eastern Central		*Dicologlossa cuneata*	2/50	25
FAO 34 Atlantic, Eastern Central	*12°50'N17°25'W*	*Galeoides decadactylus*	5/50	25
FAO 34 Atlantic, Eastern Central	*13°00'N17°15'W*	*Trachurus trecae*	2/60	50
FAO 41 Atlantic, Southwest		*Atlantoraja castelanui*	8/50	10
FAO 41 Atlantic, Southwest		*Caelorinchus fasciatus*	1/50	25
FAO 41 Atlantic, Southwest		*Genypterus blacoides*	3/50	25
FAO 41 Atlantic, Southwest		*Illex argentinus*	3/50	10
FAO 41 Atlantic, Southwest		*Macruronus magellanicus*	9/>200	28
FAO 41 Atlantic, Southwest		*Merluccius hubbsi*	6/50	25
FAO 41 Atlantic, Southwest		*Micromesistius australis*	3/50	17
FAO 41 Atlantic, Southwest		*Patagonotothen ramsayi*	1/50	25
FAO 41 Atlantic, Southwest		*Salilota australis*	2/50	25
FAO 47 Atlantic, Southeast	*13°37,09S 12°17,38'E*	*Lepidopus caudatus*	1/25	9
FAO 47 Atlantic, Southeast	*23°21,5' S 13°22,3'E*	*Merluccius capensis*	1/36	25
FAO 47 Atlantic, Southeast	*27°11,2`' S 14° 22,5'E*	*Merluccius paradoxus*	1/50	34
FAO 47 Atlantic, Southwest	*11°48,84S 13°22,97'E*	*Merluccius polli*	12/>250	30
FAO 47 Atlantic, Southeast	*25°53,1' S 13° 41,7W*	*Serranus cabrilla*	1/50	25
FAO 47 Atlantic, Southeast	*27°03,8S 14°14,7E*	*Todarodes angolensis*	3/50	10
FAO 47 Atlantic, Southeast	*24°10,9S 13°31,0'E*	*Trachurus trachurus*	2/50	22

Moreover, the different spatial distribution in fish body of *Anisakis* infecting the same fish species could be influenced by *Anisakis* species. Cipriani et al. [7] noted that in *M. merluccius* from FAO area 27, *A. simplex* larvae outnumbers *A. pegreffii* larvae in the flesh of the same fish host; on the other hand, in the viscera the mean abundance of two larvae species was superimposable. This phenomenon could be the result of different resource utilization or linked to the different migrating ability of the *Anisakis* species because of different abilities of the two species to respond to the fish host’s immune system [39].

### Safety and quality considerations

Our study confirms the presence of anisakid species with public health implications in lots of fishery products from different FAO areas. Although freezing condition and other treatments as salting and spicing assure no viable larvae in the fish products [40–43], the risk of allergens in the edible part of fish for hypersensitive individuals should be highlighted. EU legislation [44] recognizes that any parasitized fish under a visual inspection scheme should be unfit for human consumption. Comparing predictive schemes and visual inspection, in general the highest scores were associated with the acceptance of the fish batches as stated by the EU legislation. A different situation was found in the case of some batches: 7 fish sub-lots reached SADE score 4, corresponding to a “fair” FPR standard. “Fair” fish batches have neither pathological nor commercial problems (A2 SADE code – Table 4) and FBO have the possibility to give different final destinations to these fish lots, as processing, assuring safety and cost saving. Under visual inspection 4 “fair” fish batches were rejected because of the number of parasites detected (MA over 3, high parasite density – D0 SADE code) despite the absence of flesh alterations. This approach matches the precautionary principle set by Reg. EU 178/02 [20] but was restrictive in terms of economics gain. As stated by EU Reg. 853/04, FBO have to ensure that the product to be presented to the consumer is not obviously contaminated with parasites by visual inspection. Since there were different interpretations of concepts like “viable parasite” and “obviously contaminated” [16, 44–46] with a lack of standard regarding the maximum parasite limit allowed in a fish, the implementation of this risk-based surveillance according to the system developed by Llarena-Reino et al. [22] matching SADE scheme with FPR standards, should make it easier to categorize the public health and economic risk of anisakids in the flesh of commercial fish.

In this study the highest rejection rate of fish lots was under visual inspection, penalizing in some cases the FBO. SADE/FPR rejection was higher than visual inspection only in a few cases, especially for products heavily infected with deep embed larvae in which parasites were difficult to detect because of the fat percentage and colour of the viscera. However, this should be important for fish industry: in our findings, in fact, among lots with low scores, there are several fish species used for processed products of high value. In fact, *M. dypterygia* is used for deep or light salted products and *M. hubbsi*, *M. capensis* and *M. paradoxus* are the most used species for fish sticks. According to EU legislation [16], *M. paradoxus* would not have been rejected because of the low number of deep embed larvae not detectable by naked eye inspection. These sub lots, according to the scheme adopted, were rejected to prevent food business operator to suffer serious commercial losses.

This work aims to present the application of the above mentioned method on fishery products coming from several Atlantic FAO areas. Recently Rodriguez et al. [23], according to the SADE/FPR scheme, examined fish caught from 3 different ICES areas (ICES VII – Grand Sole, ICES VIII – Galician coast and ICES IX – Portuguese coast) located in the same FAO area (NE Atlantic areas – FAO 27). These authors gave “poor” FPR score to only 2 fish species, *M. merluccius* (ICES VII and ICES VIII) and *Lophius budegassa* (ICES VII), of the 9 examined, differently from our results where several fish species (*A. carbo, Z. faber, C. conger, M. dypterygia),* caught in NE Atlantic areas, reached SADE 1 and consequently “poor” FPR score.

The combined scoring systems are less restrictive than visual inspection: results compared between the different methods could be helpful to analyse an appropriate balance in terms of consumer’s safety and FBO interests. The SADE/FPR method has an acceptable sensitivity (66.7%; CI95% 34.8 – 90.1%) but a high specificity (95.2%; CI95% 76.2 – 99.9%). The accuracy of 84.85% (CI95% 68.1 – 94.9%) indicates that the SADE/FPR method has a high capacity to correctly classify fishery products. This predictive scheme, proposing corrective measures within HACCP procedures, proved to be very useful for fish lots with the lowest FPR rating particularly and offers a crucial food safety device for assessing risks associated with parasites.

## Conclusion

SADE score combined with FPR standard may represent a specific low-cost tool in fish inspection, ensuring both safety and quality, that could be useful for competent authorities and fish industry operators to establish standard management strategies. The categorization of lots in 5 quality batches, allowing the possibility of calculating accurately both parasitic load and flesh integrity, could give a unique language and *modus operandi* during self-control inspections in HACCP procedures and programs addressing fish lots in different ways depending on the score. The high specificity and accuracy of the applied predictive tests guarantees its correct applicability during the fish inspection procedures.

## Methods

### Sampling

**Fig. 1:**
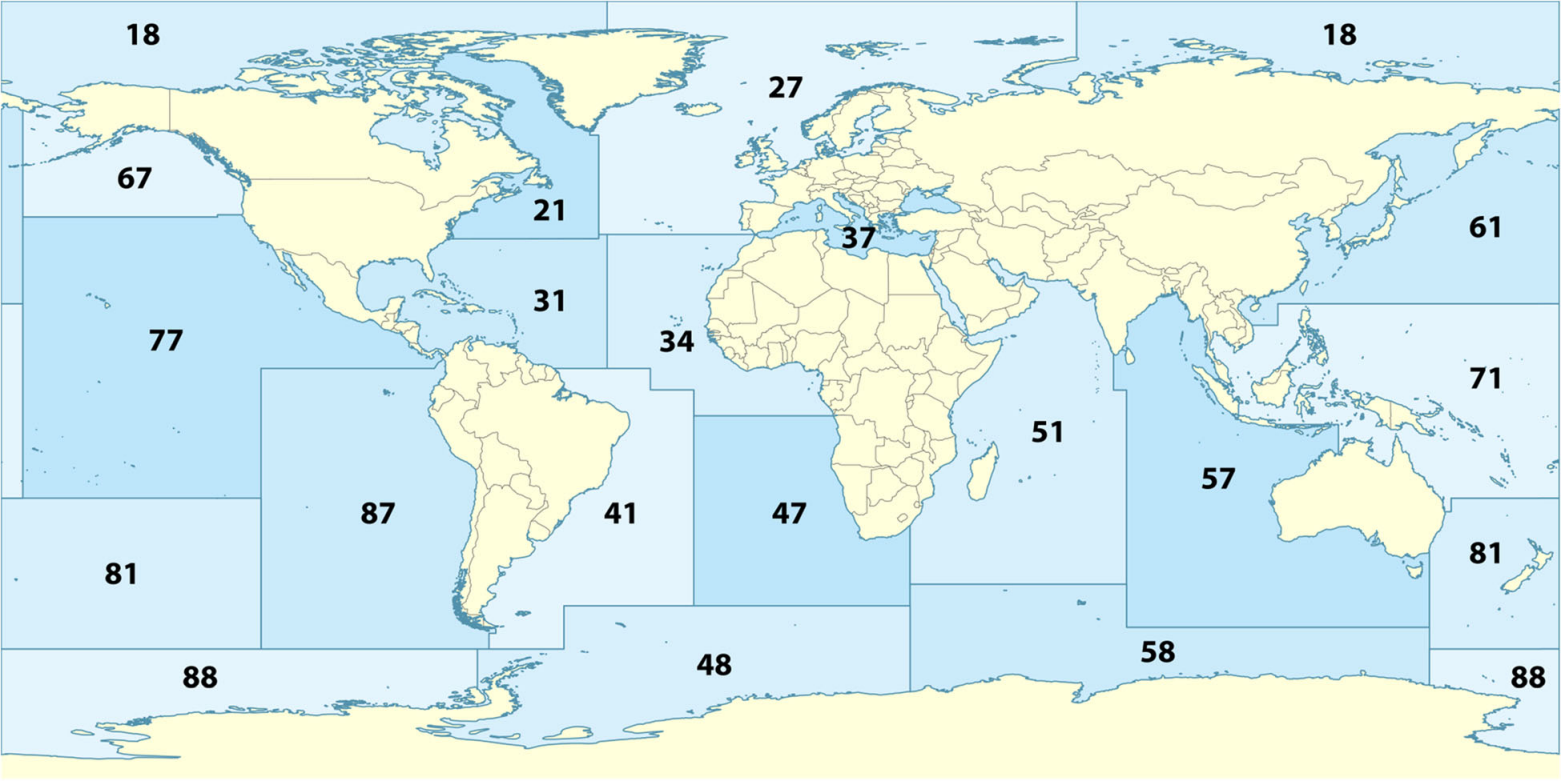
Global map of FAO Major Fishing Areas. (https://commons.wikimedia.org/w/index.php?search=fao+areas&title=Special%3ASearch&go=Go&ns0=1&ns6=1&ns12=1&ns14=1&ns100=1&ns106=1#/media/File:FAO_Major_Fishing_Areas.svg)

Between May and October 2013, a total of 771 fish and cephalopods frozen samples belonging to several commercial frozen lots (33 different species) from 5 different Atlantic FAO fishing areas (Table 5) were examined in the laboratories of the Instituto de Investigaciones Marinas de Vigo – Ecobiomar Department. FAO Fishing areas (Fig. 1) where fishery products were sampled as FAO 21 (Atlantic, Northwest, N= 130 individuals – 5 different species), FAO 27 (Atlantic, Northeast, N= 196 individuals – 9 different species), FAO 34 (Atlantic, Eastern Central, N= 100 individuals – 3 different species), FAO 41(Atlantic, Southwest, N= 190 individuals – 9 different species) and FAO 47 (Atlantic, Southwest, N= 139 individuals – 7 different species). According to Reg. EC 2074/05, a representative number of samples underwent visual inspection; for each lot (number of fish/box and number of boxes harvested by vessels that compose the lot is shown in Table 5) a representative sample (sub lot) was taken, ranging from 12% for *Merluccius polli* to 100% for *Aphanopus carbo*).

### Visual inspection

After thawing, each sub lot was weighed, each sample was weighed individually, and the abdominal cavity of each sample was opened and kept under a down-light source. The samples were gutted and the celomatic cavity was briefly inspected by the naked eye, for the presence of nematodes. According to EFSA recommendations [8], the presence of potentially zoonotic anisakid species was evaluated only in the edible parts of fishery products that is considered the main exposure risk factor for the consumer. Guts are usually discarded during fish-processing procedures. The presence of parasites on visceral peritoneum was checked but not considered a serious factor because the freezing condition prevents the migration of parasites in the flesh. For this reason, viscera were not analyzed, only the edible part of the products were considered.

### Artificial peptic digestion

After visual inspection heads and tails were removed. The remaining musculature was dissected in left and right fillets and then separated into the hypaxial (ventral) and epaxial (dorsal) regions following the horizontal septum. In case of cephalopods, the body cavity was opened and a macroscopic visual inspection was carried out.

The whole muscle (hypaxial and epaxial regions separately) of each fish sample was digested in an ACM-11806 Magnetic Stirrer Multiplate in pepsin solution [47]. For cephalopods, the mantle muscle was used to perform the process. Digestions were performed for 30 minutes at incubation temperature of 37° C in an acid solution (pH = 1.5) with HCl 0.063 M. Assays using liquid pepsin at concentration of 0.5 % and a ratio 1:20 sample weight/solution volume were used. The digestion solution was decanted through a sieve and the rests of digestion and nematodes were inspected under stereomicroscope. All anisakids were placed in individual eppendorf with ethanol 70% for further molecular diagnosis.

### Molecular analysis

All anisakid larvae were identified at genus level by microscopic examination of diagnostic characters. The biomolecular identification was performed by randomly choosing 15 larvae per species; in sub lots/species with a number of parasites lower than 15, all larvae were analyzed. A total of 275 anisakid larvae, previously identified at genus level, were used for molecular identification but only 194 were correctly classified by biomolecular analysis (Table 1). DNA extractions were performed using the commercial kit NucleoSpin®Tissue kit (Macherey-Nagel) following the manufacturer’s recommended protocols. DNA quality and quantity were checked in a spectrophotometer Nanodrop® ND-1000 (Nanodrop technologies, Inc). The entire ITS (ITS1, 5.8S rDNA gene and ITS2) was amplified using the forward primer NC5 (5’-GTA GGT GAA CCT GCG GAA GGA TCA TT-3’) and the reverse primer NC2 (5’-TTA GTT TCT TTT CCT CCG CT-3’). PCR assays were carried out in a total volume of 25 μl containing 100 ng of genomic DNA, 0.3 μM of each primer, 2.5 μl of 10x buffer, 1.5 mM of MgCl_2_, 0.2 mM of dNTPs and 0.625 U of Taq DNA polymerase (Roche Mannheim, Germany). PCR cycling parameters included denaturation at 94°C for 2 min, followed by 35 cycles of 94 °C for 30 s, annealing at 55 °C for 30 s, and extension at 72 °C for 75 s, and a final extension at 72 °C for 7 min. PCR products were purified for sequencing using ExoSAP-IT © following recommended protocol by the manufacturer. Sequencing was performed by Secugen (Madrid, Spain) and the electropherograms were analysed using the program ChromasPro version 1.41 Technelysium Pty LtdA. All sequences were searched for similarity using BLAST (Basic Local Alignment Search Tool) through web servers of the National Center for Biotechnology Information (USA).

### Risk categorisation

**Fig. 2:**
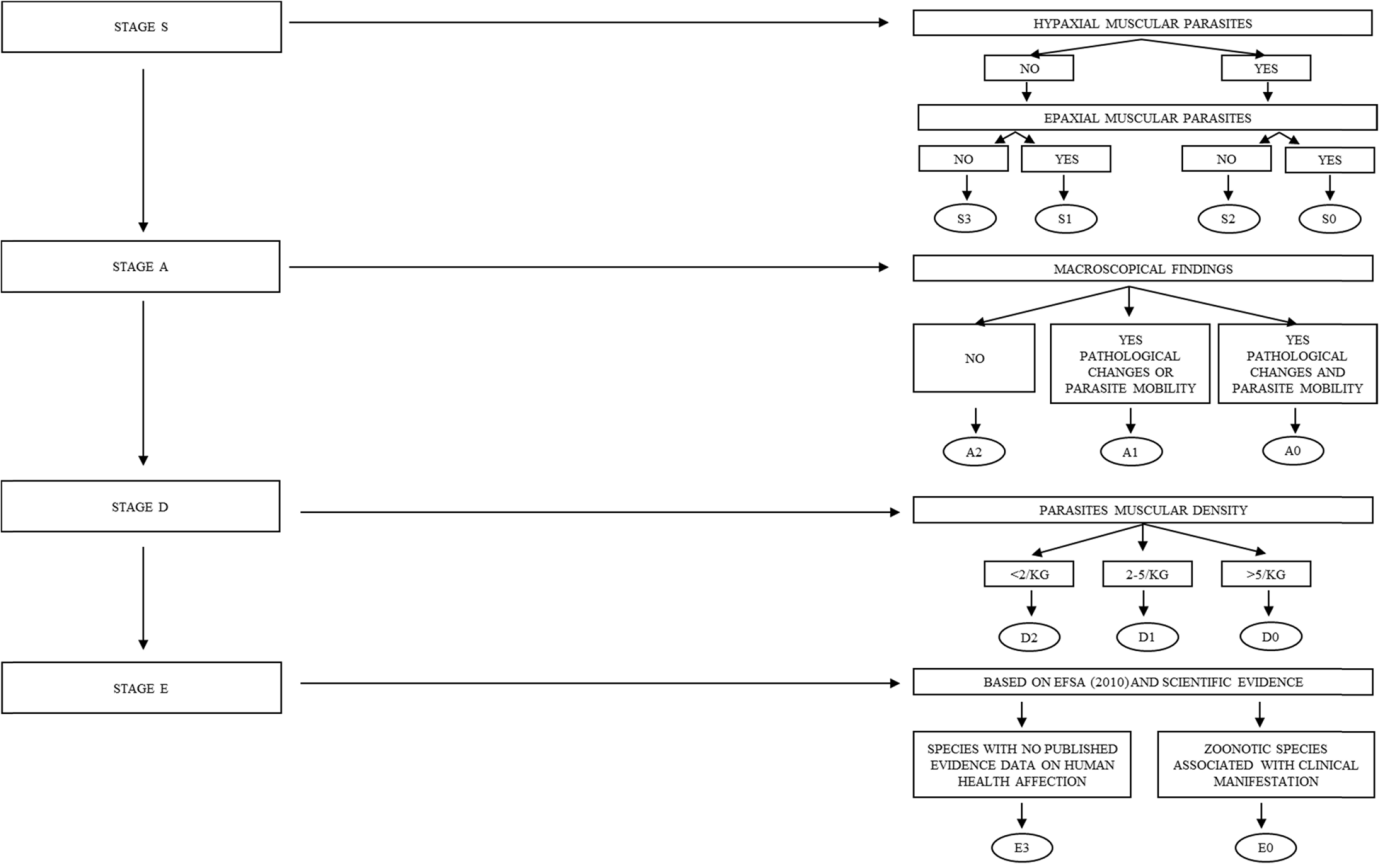
Flow diagram for the Site of infection, Assurance of quality, Demography, Epidemiology (SADE) modified according to Llarena-reino et al., 2013

Briefly sub lots are grouped according to four homogeneous categories named S, A, D, and E, that are afterwards split into numeric subcategories by the means of a flow diagram (Fig. 2 modified according to Llarena-reino et al., [22]): each inspected fish lot was categorized according to the localization of parasites (S – hypaxial or epaxial musculature flesh), the presence/absence of pathological or unaesthetic signs in the edible part (A), the density of infection (D – number of larvae/kg of fish) and finally the epidemiological relevance of the parasites (E – zoonotic or not). By adding the numeric values of each categories, the SADE system adopts a 10-point scale: as a result, a SADE code and a final score are obtained for each lot checked, in order to decide the final destination.

The highest values indicate no risk connected to the examined lots, whereas the lowest score suggests serious issues connected to fish parasites.

**Fig. 3:**
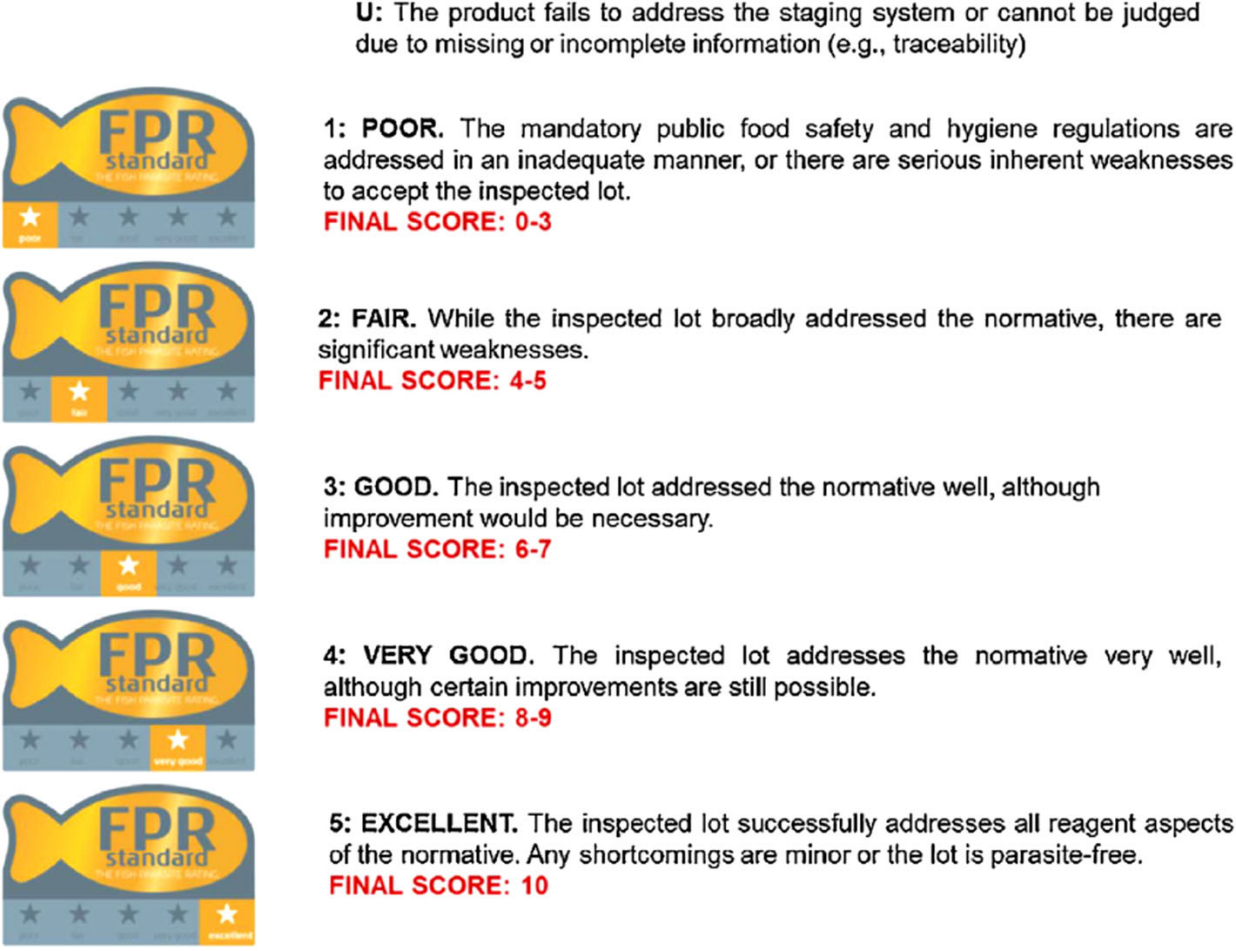
FPR (Fish Parasite Rating) standard categories, based in the scoring system approach (SADE). Rodríguez et al., 2017

These preliminary results were translated to the standard Fish Parasite Rating (FPR) score, which allows the classification of fish lots into five categories (Fig. 3):

- Poor: final score 0-3;
- Fair: final score 4-5;
- Good: final score 6-7
- Very good: final score 8-9;
- Excellent: final score 10.

Since the SADE scheme is used for the evaluation of homogenous commercial fish lots, the scientists who took part in the study did not proceed to the individual measurement of the sample. The products were only weighed in order to assess the parasitic density (n. Parasites / kg).

Finally, according to the European Hygiene Rules (Reg. EC 853/2004, Section VIII, Chapter V, Pt. D), the percentage of fishery products that should be unfit for human consumption was calculated and compared with SADE – FPD scores.

### Statistical analysis

To describe parasite population the following parameters were used, according to Bush et al. [24]: P (the ratio between parasitized subjects/sub lots and the total subjects/ sub lots analysed), MA (the ratio between the number of larvae recovered and the number of all examined subjects of sub lots) and MI (the ratio between the number of larvae recovered and the number of examined parasitized subjects of sub lots). Infection indexes were calculated regardless of parasites’ localisation site (Epaxial/Hypaxial flesh), species/sub lot and for each FAO areas. The differences in the P between FAO areas were assessed by the two-sided chi-square test.

Statistical significance between MA and MI of different FAO Areas was performed using One-way ANOVA with Bonferroni’s post-test.

In order to assess statistical association between MA and fish sample mean weight a simple regression analysis was used. Finally, statistical comparisons between SADE/FPR and Visual Inspection were performed by McNemar’s chi-square test [48]. Moreover, sensitivity, specificity and accuracy values of the SADE/FPR relative to Visual Inspection were calculated. Statistical analyses were performed using GraphPad InStat Version 3.0 (GraphPad Software, San Diego California USA) and MedCalc for Windows, version 18.11.3 (MedCalc Software, Ostend, Belgium); *p* < 0.05 was considered significant for all statistical tests.

## Supplementary information

**Additional file 1.** Identified parasites in fish species,Accession ID related to the aligned sequences and web links (https://www.ncbi.nlm.nih.gov/pubmed/).

## Data Availability

The datasets used and/or analyzed during the current study are available from the corresponding author on reasonable request. Sequencing was performed by Secugen (Madrid, Spain) and the electropherograms were analysed using the program ChromasPro version 1.41 Technelysium Pty LtdA. All sequences were searched for similarity using BLAST (Basic Local Alignment Search Tool) (https://blast.ncbi.nlm.nih.gov/Blast.cgi). Accession ID were in the supplementary materials.

## Abbreviations

EFSA: European Food Safety Authority
EU: European Union
EU: European Union
FAO: Food and Agriculture Organization of the United Nations
FBO: Food Business Operators
FPR: Fish Parasite Rating
HACCP: Hazard Analysis and Critical Control Points
ICES: International Council for the Exploration of the Sea
MA: Mean abundance
MI: Mean intensity
OHIM: Office for Harmonization in the Internal Market
P: Prevalence of infection
RASFF: Rapid Alert System for Food and Feed
SADE: Site, abundance, density and epidemiology
