# Supplementary material for: Risk-based scoring and genetic identification for anisakids in frozen fish products from Atlantic FAO areas
Source: BMC Vet Res. 2020 Feb 21;16:65. doi: 10.1186/s12917-020-02286-7 (PMC7033913; doi:10.1186/s12917-020-02286-7)
Supplement: Supplementary file 1 — Additional file 1. Identified parasites in fish species,Accession ID related to the aligned sequences and web links (https://www.ncbi.nlm.nih.gov/pubmed/). [file 12917_2020_2286_MOESM1_ESM.docx]

**Supplementary materials**

Identified parasites in fish species,Accession ID related to the aligned sequences and web links (https://www.ncbi.nlm.nih.gov/pubmed/)

| ***Fao Areas*** | ***Identified Parasites*** | ***Accession ID*** | ***Web link*** | ***Fish Species*** |
| --- | --- | --- | --- | --- |
| FAO 21 | *A. simplex* | KC121369.1 | <https://www.ncbi.nlm.nih.gov/nuccore/445065176/> | *Macrurus Berglax* |
| FAO 21 | *A. simplex* | JX237370.1 | https://www.ncbi.nlm.nih.gov/nuccore/403492157/ | *Macrurus Berglax* |
| FAO 21 | *A. simplex* | JX237370.1 | https://www.ncbi.nlm.nih.gov/nuccore/403492157/ | *Macrurus Berglax* |
| FAO 21 | *A. simplex* | JX237370.1 | https://www.ncbi.nlm.nih.gov/nuccore/403492157/ | *Macrurus Berglax* |
| FAO 21 | *A. simplex* | HQ717429.1 | https://www.ncbi.nlm.nih.gov/nuccore/327201253/ | *Macrurus Berglax* |
| FAO 21 | *A. simplex* | JX237370.1 | https://www.ncbi.nlm.nih.gov/nuccore/403492157/ | *Macrurus Berglax* |
| FAO 21 | *A. simplex* | HQ717429.1 | https://www.ncbi.nlm.nih.gov/nuccore/327201253/ | *Macrurus Berglax* |
| FAO 21 | *A. simplex* | EU624342.1 | https://www.ncbi.nlm.nih.gov/nuccore/186920351/ | *Macrurus Berglax* |
| FAO 21 | *A. simplex* | JX237370.1 | https://www.ncbi.nlm.nih.gov/nuccore/403492157/ | *Macrurus Berglax* |
| FAO 21 | *A. simplex* | JX237370.1 | https://www.ncbi.nlm.nih.gov/nuccore/403492157/ | *Macrurus Berglax* |
| FAO 21 | *A. simplex* | JN968705.1 | https://www.ncbi.nlm.nih.gov/nuccore/JN968705.1 | *Macrurus Berglax* |
| FAO 21 | *A. simplex* | JX237370.1 | https://www.ncbi.nlm.nih.gov/nuccore/403492157/ | *Macrurus Berglax* |
| FAO 21 | *A. simplex* | JX237370.1 | https://www.ncbi.nlm.nih.gov/nuccore/403492157/ | *Macrurus Berglax* |
| FAO 21 | *A. simplex* | JX237370.1 | https://www.ncbi.nlm.nih.gov/nuccore/403492157/ | *Reinharditius Hippoglossoides* |
| FAO 21 | *A. simplex* | JX237370.1 | https://www.ncbi.nlm.nih.gov/nuccore/403492157/ | *Reinharditius Hippoglossoides* |
| FAO 21 | *A. simplex* | JX237370.1 | https://www.ncbi.nlm.nih.gov/nuccore/403492157/ | *Reinharditius Hippoglossoides* |
| FAO 21 | *A. simplex* | JX237370.1 | https://www.ncbi.nlm.nih.gov/nuccore/403492157/ | *Reinharditius Hippoglossoides* |
| FAO 21 | *A. simplex* | JN968705.1 | https://www.ncbi.nlm.nih.gov/nuccore/JN968705.1 | *Reinharditius Hippoglossoides* |
| FAO 21 | *A. simplex* | JX237370.1 | https://www.ncbi.nlm.nih.gov/nuccore/403492157/ | *Reinharditius Hippoglossoides* |
| FAO 21 | *A. simplex* | EU624342.1 | <https://www.ncbi.nlm.nih.gov/nuccore/EU624342.1> | *Reinharditius Hippoglossoides* |
| FAO 21 | *A. simplex* | HQ717429.1 | <https://www.ncbi.nlm.nih.gov/nuccore/327201253/> | *Reinharditius Hippoglossoides* |
| FAO 21 | *A. simplex* | JX237370.1 | https://www.ncbi.nlm.nih.gov/nuccore/403492157/ | *Reinharditius Hippoglossoides* |
| FAO 21 | *A. simplex* | JX237370.1 | https://www.ncbi.nlm.nih.gov/nuccore/403492157/ | *Reinharditius Hippoglossoides* |
| FAO 21 | *A. simplex* | JX237370.1 | https://www.ncbi.nlm.nih.gov/nuccore/403492157/ | *Reinharditius Hippoglossoides* |
| FAO 21 | *A. simplex* | AJ937670.1 | <https://www.ncbi.nlm.nih.gov/nuccore/AJ937670.1> | *Reinharditius Hippoglossoides* |
| FAO 21 | *A. simplex* | JX237370.1 | https://www.ncbi.nlm.nih.gov/nuccore/403492157/ | *Reinharditius Hippoglossoides* |
| FAO 21 | *A. simplex* | JX237370.1 | https://www.ncbi.nlm.nih.gov/nuccore/403492157/ | *Reinharditius Hippoglossoides* |
| FAO 21 | *A. simplex* | AJ937670.1 | <https://www.ncbi.nlm.nih.gov/nuccore/AJ937670.1> | *Urophycis Chus* |
| FAO 21 | *A. simplex* | JX237370.1 | https://www.ncbi.nlm.nih.gov/nuccore/403492157/ | *Urophycis Chus* |
| FAO 21 | *A. simplex* | JX237370.1 | https://www.ncbi.nlm.nih.gov/nuccore/403492157/ | *Urophycis Chus* |
| FAO 21 | *A. simplex* | JX237370.1 | https://www.ncbi.nlm.nih.gov/nuccore/403492157/ | *Urophycis Chus* |
| FAO 21 | *A. simplex* | JX237370.1 | https://www.ncbi.nlm.nih.gov/nuccore/403492157/ | *Urophycis Chus* |
| FAO 21 | *A. simplex* | JX237370.1 | https://www.ncbi.nlm.nih.gov/nuccore/403492157/ | *Urophycis Chus* |
| FAO 21 | *A. simplex* | HQ717429.1 | <https://www.ncbi.nlm.nih.gov/nuccore/327201253/> | *Urophycis Chus* |
| FAO 27 | *A. simplex* | JX237370.1 | https://www.ncbi.nlm.nih.gov/nuccore/403492157/ | *Alepocephalus Bairdii* |
| FAO 27 | *A. simplex* | JX237370.1 | https://www.ncbi.nlm.nih.gov/nuccore/403492157/ | *Alepocephalus Bairdii* |
| FAO 27 | *A. simplex* | JN968834.1 | <https://www.ncbi.nlm.nih.gov/nuccore/JN968705.1> | *Alepocephalus Bairdii* |
| FAO 27 | *A. simplex* | JX237370.1 | https://www.ncbi.nlm.nih.gov/nuccore/403492157/ | *Alepocephalus Bairdii* |
| FAO 27 | *A. simplex* | JX237370.1 | https://www.ncbi.nlm.nih.gov/nuccore/403492157/ | *Alepocephalus Bairdii* |
| FAO 27 | *A. simplex* | GQ169362.1 | <https://www.ncbi.nlm.nih.gov/nuccore/GQ169362.1> | *Zeus Faber* |
| FAO 27 | *A. simplex* | JX237370.1 | https://www.ncbi.nlm.nih.gov/nuccore/403492157/ | *Zeus Faber* |
| FAO 27 | *A. simplex* | EU624342.1 | <https://www.ncbi.nlm.nih.gov/nuccore/EU624342.1> | *Zeus Faber* |
| FAO 27 | *A. simplex* | JX237370.1 | https://www.ncbi.nlm.nih.gov/nuccore/403492157/ | *Zeus Faber* |
| FAO 27 | *A. simplex* | EU624342.1 | <https://www.ncbi.nlm.nih.gov/nuccore/EU624342.1> | *Zeus Faber* |
| FAO 27 | *A. simplex* | GQ169362.1 | <https://www.ncbi.nlm.nih.gov/nuccore/GQ169362.1> | *Zeus Faber* |
| FAO 27 | *A. simplex* | GQ169362.1 | <https://www.ncbi.nlm.nih.gov/nuccore/GQ169362.1> | *Zeus Faber* |
| FAO 27 | *P. decipiens s. l.* | JQ673263.1 | <https://www.ncbi.nlm.nih.gov/nuccore/JQ673263.1> | *Zeus Faber* |
| FAO 27 | *A. simplex* | JX237370.1 | https://www.ncbi.nlm.nih.gov/nuccore/403492157/ | *Zeus Faber* |
| FAO 27 | *A. simplex* | JX237370.1 | https://www.ncbi.nlm.nih.gov/nuccore/403492157/ | *Zeus Faber* |
| FAO 27 | *A. simplex* | GQ169362.1 | <https://www.ncbi.nlm.nih.gov/nuccore/GQ169362.1> | *Zeus Faber* |
| FAO 27 | *A. simplex* | JX237370.1 | https://www.ncbi.nlm.nih.gov/nuccore/403492157/ | *Aphanopus Carbo* |
| FAO 27 | *A. simplex* | JX237370.1 | https://www.ncbi.nlm.nih.gov/nuccore/403492157/ | *Aphanopus Carbo* |
| FAO 27 | *A. simplex* | JX237370.1 | https://www.ncbi.nlm.nih.gov/nuccore/403492157/ | *Aphanopus Carbo* |
| FAO 27 | *A. simplex* | JX237370.1 | https://www.ncbi.nlm.nih.gov/nuccore/403492157/ | *Aphanopus Carbo* |
| FAO 27 | *A. simplex* | GQ169362.1 | <https://www.ncbi.nlm.nih.gov/nuccore/GQ169362.1> | *Aphanopus Carbo* |
| FAO 27 | *A. simplex* | JX237370.1 | https://www.ncbi.nlm.nih.gov/nuccore/403492157/ | *Aphanopus Carbo* |
| FAO 27 | *A. pegreffii* | KF032066.1 | <https://www.ncbi.nlm.nih.gov/nuccore/KF032066.1> | *Regalecus Glesne* |
| FAO 27 | *A. simplex* | EU624342.1 | <https://www.ncbi.nlm.nih.gov/nuccore/EU624342.1> | *Regalecus Glesne* |
| FAO 27 | *A. simplex* | JN968705.1 | <https://www.ncbi.nlm.nih.gov/nuccore/JN968705.1> | *Regalecus Glesne* |
| FAO 27 | *A. simplex* | EU624342.1 | <https://www.ncbi.nlm.nih.gov/nuccore/EU624342.1> | *Molva Dypterygia* |
| FAO 27 | *A. simplex* | JX237370.1 | https://www.ncbi.nlm.nih.gov/nuccore/403492157/ | *Molva Dypterygia* |
| FAO 27 | *A. simplex* | JX237370.1 | https://www.ncbi.nlm.nih.gov/nuccore/403492157/ | *Molva Dypterygia* |
| FAO 27 | *A. simplex* | JX237370.1 | https://www.ncbi.nlm.nih.gov/nuccore/403492157/ | *Molva Dypterygia* |
| FAO 27 | *A. simplex* | JX237370.1 | https://www.ncbi.nlm.nih.gov/nuccore/403492157/ | *Molva Dypterygia* |
| FAO 27 | *A. simplex* | JX237370.1 | https://www.ncbi.nlm.nih.gov/nuccore/403492157/ | *Molva Dypterygia* |
| FAO 27 | *A. simplex* | JX237370.1 | https://www.ncbi.nlm.nih.gov/nuccore/403492157/ | *Molva Dypterygia* |
| FAO 27 | *A. simplex* | GQ169362.1 | <https://www.ncbi.nlm.nih.gov/nuccore/GQ169362.1> | *Molva Dypterygia* |
| FAO 27 | *A. simplex* | JX237370.1 | https://www.ncbi.nlm.nih.gov/nuccore/403492157/ | *Trachyrhynchus Scabrus* |
| FAO 27 | *A. simplex* | JX237370.1 | https://www.ncbi.nlm.nih.gov/nuccore/403492157/ | *Trachyrhynchus Scabrus* |
| FAO 27 | *A. simplex* | JX237370.1 | https://www.ncbi.nlm.nih.gov/nuccore/403492157/ | *Trachyrhynchus Scabrus* |
| FAO 27 | *A. simplex* | JX237370.1 | https://www.ncbi.nlm.nih.gov/nuccore/403492157/ | *Trachyrhynchus Scabrus* |
| FAO 27 | *A. simplex* | JX237370.1 | https://www.ncbi.nlm.nih.gov/nuccore/403492157/ | *Trachyrhynchus Scabrus* |
| FAO 27 | *A. simplex* | JX237370.1 | https://www.ncbi.nlm.nih.gov/nuccore/403492157/ | *Trachyrhynchus Scabrus* |
| FAO 27 | *A. simplex* | JX237370.1 | https://www.ncbi.nlm.nih.gov/nuccore/403492157/ | *Melanogrammus Aeglefinus* |
| FAO 27 | *A. simplex* | JX237370.1 | https://www.ncbi.nlm.nih.gov/nuccore/403492157/ | *Melanogrammus Aeglefinus* |
| FAO 27 | *A. simplex* | JX237370.1 | https://www.ncbi.nlm.nih.gov/nuccore/403492157/ | *Melanogrammus Aeglefinus* |
| FAO 27 | *A. simplex* | EU624342.1 | <https://www.ncbi.nlm.nih.gov/nuccore/EU624342.1> | *Melanogrammus Aeglefinus* |
| FAO 27 | *A. simplex* | JX237370.1 | https://www.ncbi.nlm.nih.gov/nuccore/403492157/ | *Melanogrammus Aeglefinus* |
| FAO 27 | *P. decipiens s. l.* | JQ673263.1 | <https://www.ncbi.nlm.nih.gov/nuccore/JQ673263.1> | *C.Conger* |
| FAO 27 | *A. simplex* | JX237370.1 | https://www.ncbi.nlm.nih.gov/nuccore/403492157/ | *C.Conger* |
| FAO 27 | *A. simplex* | JX237370.1 | https://www.ncbi.nlm.nih.gov/nuccore/403492157/ | *C.Conger* |
| FAO 27 | *P. decipiens s. l.* | JQ673263.1 | <https://www.ncbi.nlm.nih.gov/nuccore/JQ673263.1> | *C.Conger* |
| FAO 27 | *P. decipiens s. l.* | JQ673263.1 | <https://www.ncbi.nlm.nih.gov/nuccore/JQ673263.1> | *C.Conger* |
| FAO 27 | *P. decipiens s. l.* | JQ673263.1 | <https://www.ncbi.nlm.nih.gov/nuccore/JQ673263.1> | *C.Conger* |
| FAO 27 | *A. simplex* | JX237370.1 | https://www.ncbi.nlm.nih.gov/nuccore/403492157/ | *C.Conger* |
| FAO 27 | *A. simplex* | JX237370.1 | https://www.ncbi.nlm.nih.gov/nuccore/403492157/ | *C.Conger* |
| FAO 27 | *A. simplex* | JX237370.1 | https://www.ncbi.nlm.nih.gov/nuccore/403492157/ | *C.Conger* |
| FAO 27 | *P. decipiens s. l.* | JX138340.1 | <https://www.ncbi.nlm.nih.gov/nuccore/JX138340.1> | *C.Conger* |
| FAO 27 | *A. pegreffii* | KF032066.1 | <https://www.ncbi.nlm.nih.gov/nuccore/KF032066.1> | *C.Conger* |
| FAO 27 | *A. simplex* | JX237370.1 | https://www.ncbi.nlm.nih.gov/nuccore/403492157/ | *C.Conger* |
| FAO 27 | *A. simplex* | JX237370.1 | https://www.ncbi.nlm.nih.gov/nuccore/403492157/ | *Clupea Harengus* |
| FAO 27 | *A. simplex* | JN968834.1 | <https://www.ncbi.nlm.nih.gov/nuccore/JN968834.1> | *Clupea Harengus* |
| FAO 27 | *A. simplex* | JX237370.1 | https://www.ncbi.nlm.nih.gov/nuccore/403492157/ | *Clupea Harengus* |
| FAO 27 | *A. simplex* | GQ169363.1 | <https://www.ncbi.nlm.nih.gov/nuccore/GQ169362.1> | *Clupea Harengus* |
| FAO 27 | *A. simplex* | JX237370.1 | https://www.ncbi.nlm.nih.gov/nuccore/403492157/ | *Clupea Harengus* |
| FAO 27 | *A. simplex* | JX237370.1 | https://www.ncbi.nlm.nih.gov/nuccore/403492157/ | *Clupea Harengus* |
| FAO 27 | *A. simplex* | GQ472924.1 | <https://www.ncbi.nlm.nih.gov/nuccore/GQ472924.1> | *Clupea Harengus* |
| FAO 27 | *A. simplex* | JX237370.1 | https://www.ncbi.nlm.nih.gov/nuccore/403492157/ | *Clupea Harengus* |
| FAO 27 | *A. simplex* | GQ169362.1 | <https://www.ncbi.nlm.nih.gov/nuccore/GQ169362.1> | *Clupea Harengus* |
| FAO 27 | *A. simplex* | JX237370.1 | https://www.ncbi.nlm.nih.gov/nuccore/403492157/ | *Clupea Harengus* |
| FAO 27 | *A. simplex* | JX237370.1 | https://www.ncbi.nlm.nih.gov/nuccore/403492157/ | *Clupea Harengus* |
| FAO 27 | *A. simplex* | JX237370.1 | https://www.ncbi.nlm.nih.gov/nuccore/403492157/ | *Clupea Harengus* |
| FAO 41 | *A. pegreffii* | KF032066.1 | <https://www.ncbi.nlm.nih.gov/nuccore/KF032066.1> | *Macruronus Magellanicus* |
| FAO 41 | *A. pegreffii* | KF032066.1 | <https://www.ncbi.nlm.nih.gov/nuccore/KF032066.1> | *Macruronus Magellanicus* |
| FAO 41 | *A. pegreffii* | KF032066.1 | <https://www.ncbi.nlm.nih.gov/nuccore/KF032066.1> | *Merluccius Hubbsi* |
| FAO 41 | *A. pegreffii* | KF032066.1 | <https://www.ncbi.nlm.nih.gov/nuccore/KF032066.1> | *Merluccius Hubbsi* |
| FAO 41 | *A. pegreffii* | KF032066.1 | <https://www.ncbi.nlm.nih.gov/nuccore/KF032066.1> | *Merluccius Hubbsi* |
| FAO 41 | *A. pegreffii* | KF032066.1 | <https://www.ncbi.nlm.nih.gov/nuccore/KF032066.1> | *Merluccius Hubbsi* |
| FAO 41 | *A. pegreffii* | KF032066.1 | <https://www.ncbi.nlm.nih.gov/nuccore/KF032066.1> | *Merluccius Hubbsi* |
| FAO 41 | *C. osculatum* | AB277825.1 | <https://www.ncbi.nlm.nih.gov/nuccore/AB277825.1> | *Merluccius Hubbsi* |
| FAO 41 | *A. berlandi* | JN968646.1 | <https://www.ncbi.nlm.nih.gov/nuccore/JN968646.1> | *Merluccius Hubbsi* |
| FAO 41 | *C. osculatum* | AB277825.1 | <https://www.ncbi.nlm.nih.gov/nuccore/AB277825.1> | *Merluccius Hubbsi* |
| FAO 41 | *A. pegreffii* | KF032066.1 | <https://www.ncbi.nlm.nih.gov/nuccore/KF032066.1> | *Merluccius Hubbsi* |
| FAO 41 | *A. pegreffii* | KF032066.1 | <https://www.ncbi.nlm.nih.gov/nuccore/KF032066.1> | *Merluccius Hubbsi* |
| FAO 41 | *A. pegreffii* | KF032066.1 | <https://www.ncbi.nlm.nih.gov/nuccore/KF032066.1> | *Merluccius Hubbsi* |
| FAO 41 | *A. pegreffii* | KF032066.1 | <https://www.ncbi.nlm.nih.gov/nuccore/KF032066.1> | *Merluccius Hubbsi* |
| FAO 41 | *A. pegreffii* | KF032066.1 | <https://www.ncbi.nlm.nih.gov/nuccore/KF032066.1> | *Merluccius Hubbsi* |
| FAO 41 | *C. osculatum* | AB277825.1 | <https://www.ncbi.nlm.nih.gov/nuccore/AB277825.1> | *Merluccius Hubbsi* |
| FAO 41 | *A. pegreffii* | KF032066.1 | <https://www.ncbi.nlm.nih.gov/nuccore/KF032066.1> | *Micromesistius Australis* |
| FAO 41 | *A. berlandi* | KC121371.1 | <https://www.ncbi.nlm.nih.gov/nuccore/KC121371.1> | *Micromesistius Australis* |
| FAO 41 | *A. pegreffii* | KF032066.1 | <https://www.ncbi.nlm.nih.gov/nuccore/KF032066.1> | *Micromesistius Australis* |
| FAO 41 | *A. pegreffii* | KF032066.1 | <https://www.ncbi.nlm.nih.gov/nuccore/KF032066.1> | *Micromesistius Australis* |
| FAO 41 | *A. pegreffii* | KF032066.1 | <https://www.ncbi.nlm.nih.gov/nuccore/KF032066.1> | *Micromesistius Australis* |
| FAO 41 | *A. pegreffii* | KF032066.1 | <https://www.ncbi.nlm.nih.gov/nuccore/KF032066.1> | *Micromesistius Australis* |
| FAO 41 | *C. osculatum* | AB277825.1 | <https://www.ncbi.nlm.nih.gov/nuccore/AB277825.1> | *Micromesistius Australis* |
| FAO 41 | *A. pegreffii* | KF032066.1 | <https://www.ncbi.nlm.nih.gov/nuccore/KF032066.1> | *Micromesistius Australis* |
| FAO 41 | *A. pegreffii* | KF032066.1 | <https://www.ncbi.nlm.nih.gov/nuccore/KF032066.1> | *Micromesistius Australis* |
| FAO 41 | *A. berlandi* | AY821736.1 | <https://www.ncbi.nlm.nih.gov/nuccore/AY821736.1> | *Micromesistius Australis* |
| FAO 41 | *A. berlandi* | JN968652.1 | <https://www.ncbi.nlm.nih.gov/nuccore/JN968646.1> | *Micromesistius Australis* |
| FAO 41 | *A. pegreffii* | KF032066.1 | <https://www.ncbi.nlm.nih.gov/nuccore/KF032066.1> | *Micromesistius Australis* |
| FAO 41 | *A. simplex* | GQ169362.1 | <https://www.ncbi.nlm.nih.gov/nuccore/GQ169362.1> | *Illex Argentinus* |
| FAO 41 | *H. aduncum* | JX845137.1 | <https://www.ncbi.nlm.nih.gov/nuccore/JX845137.1> | *Illex Argentinus* |
| FAO 41 | *A. pegreffii* | KF032066.1 | <https://www.ncbi.nlm.nih.gov/nuccore/KF032066.1> | *Caelorinchus Fabiatus* |
| FAO 41 | *A. berlandi* | AY821736.1 | <https://www.ncbi.nlm.nih.gov/nuccore/AY821736.1> | *Caelorinchus Fabiatus* |
| FAO 41 | *A. pegreffii* | KF032066.1 | <https://www.ncbi.nlm.nih.gov/nuccore/KF032066.1> | *Caelorinchus Fabiatus* |
| FAO 41 | *A. pegreffii* | KF032066.1 | <https://www.ncbi.nlm.nih.gov/nuccore/KF032066.1> | *G. Blacoides* |
| FAO 41 | *A. pegreffii* | KF032066.1 | <https://www.ncbi.nlm.nih.gov/nuccore/KF032066.1> | *G. Blacoides* |
| FAO 41 | *A. pegreffii* | KF032066.1 | <https://www.ncbi.nlm.nih.gov/nuccore/KF032066.1> | *G. Blacoides* |
| FAO 41 | *A. berlandi* | KC121371.1 | <https://www.ncbi.nlm.nih.gov/nuccore/KC121371.1> | *G. Blacoides* |
| FAO 41 | *A. pegreffii* | KF032066.1 | <https://www.ncbi.nlm.nih.gov/nuccore/KF032066.1> | *G. Blacoides* |
| FAO 41 | *A. pegreffii* | JN968604.1 | <https://www.ncbi.nlm.nih.gov/nuccore/JN968646.1> | *G. Blacoides* |
| FAO 41 | *P. cattani* | KF781285.1 | <https://www.ncbi.nlm.nih.gov/nuccore/KF781285.1> | *G. Blacoides* |
| FAO 41 | *A. berlandi* | JN968652.1 | <https://www.ncbi.nlm.nih.gov/nuccore/JN968646.1> | *G. Blacoides* |
| FAO 41 | *A. pegreffii* | JF683735.1 | <https://www.ncbi.nlm.nih.gov/nuccore/JF683735.1> | *G. Blacoides* |
| FAO 41 | *A. pegreffii* | KF032066.1 | <https://www.ncbi.nlm.nih.gov/nuccore/KF032066.1> | *G. Blacoides* |
| FAO 41 | *A. berlandi* | JN968652.1 | <https://www.ncbi.nlm.nih.gov/nuccore/JN968646.1> | *G. Blacoides* |
| FAO 47 | *A. berlandi* | JN968646.1 | <https://www.ncbi.nlm.nih.gov/nuccore/JN968646.1> | *Todarodes Angolensis* |
| FAO 47 | *A. berlandi* | JN968646.1 | <https://www.ncbi.nlm.nih.gov/nuccore/JN968646.1> | *Todarodes Angolensis* |
| FAO 47 | *A. berlandi* | JN968646.1 | <https://www.ncbi.nlm.nih.gov/nuccore/JN968646.1> | *Todarodes Angolensis* |
| FAO 47 | *A. typica* | KC928261.1 | <https://www.ncbi.nlm.nih.gov/nuccore/KC928261.1> | *Lepidopus caudatus* |
| FAO 47 | *A. typica* | JQ912690.1 | <https://www.ncbi.nlm.nih.gov/nuccore/JQ912690.1> | *Lepidopus caudatus* |
| FAO 47 | *A. typica* | JQ912690.1 | <https://www.ncbi.nlm.nih.gov/nuccore/JQ912690.1> | *Lepidopus caudatus* |
| FAO 47 | *A. typica* | KC928261.1 | <https://www.ncbi.nlm.nih.gov/nuccore/KC928261.1> | *Lepidopus caudatus* |
| FAO 47 | *A. typica* | KC928261.1 | <https://www.ncbi.nlm.nih.gov/nuccore/KC928261.1> | *Lepidopus caudatus* |
| FAO 47 | *A. typica* | JQ912690.1 | <https://www.ncbi.nlm.nih.gov/nuccore/JQ912690.1> | *Lepidopus caudatus* |
| FAO 47 | *A. typica* | JQ912690.1 | <https://www.ncbi.nlm.nih.gov/nuccore/JQ912690.1> | *Lepidopus caudatus* |
| FAO 47 | *A. typica* | JQ912690.1 | <https://www.ncbi.nlm.nih.gov/nuccore/JQ912690.1> | *Lepidopus caudatus* |
| FAO 47 | *A. typica* | AB479120.1 | <https://www.ncbi.nlm.nih.gov/nuccore/AB479120.1> | *Lepidopus caudatus* |
| FAO 47 | *A. pegreffii* | KF032066.1 | <https://www.ncbi.nlm.nih.gov/nuccore/KF032066.1> | *Merluccius Paradoxus* |
| FAO 47 | *A. pegreffii* | KF032066.1 | <https://www.ncbi.nlm.nih.gov/nuccore/KF032066.1> | *Merluccius Paradoxus* |
| FAO 47 | *A. pegreffii* | KF032066.1 | <https://www.ncbi.nlm.nih.gov/nuccore/KF032066.1> | *Merluccius Paradoxus* |
| FAO 47 | *A. typica* | JQ912690.1 | <https://www.ncbi.nlm.nih.gov/nuccore/JQ912690.1> | *Merluccius Paradoxus* |
| FAO 47 | *A. pegreffii* | KF032066.1 | <https://www.ncbi.nlm.nih.gov/nuccore/KF032066.1> | *Merluccius Paradoxus* |
| FAO 47 | *A. pegreffii* | KF032066.1 | <https://www.ncbi.nlm.nih.gov/nuccore/KF032066.1> | *Merluccius Paradoxus* |
| FAO 47 | *A. pegreffii* | KF032066.1 | <https://www.ncbi.nlm.nih.gov/nuccore/KF032066.1> | *Merluccius Paradoxus* |
| FAO 47 | *A. pegreffii* | KF032066.1 | <https://www.ncbi.nlm.nih.gov/nuccore/KF032066.1> | *Merluccius Paradoxus* |
| FAO 47 | *A. pegreffii* | KF032066.1 | <https://www.ncbi.nlm.nih.gov/nuccore/KF032066.1> | *Merluccius Paradoxus* |
| FAO 47 | *A. pegreffii* | KF032066.1 | <https://www.ncbi.nlm.nih.gov/nuccore/KF032066.1> | *Merluccius Paradoxus* |
| FAO 47 | *A. pegreffii* | KF032066.1 | <https://www.ncbi.nlm.nih.gov/nuccore/KF032066.1> | *Merluccius Paradoxus* |
| FAO 47 | *A. pegreffii* | KF032066.1 | <https://www.ncbi.nlm.nih.gov/nuccore/KF032066.1> | *Merluccius Paradoxus* |
| FAO 47 | *A. pegreffii* | KF032066.1 | <https://www.ncbi.nlm.nih.gov/nuccore/KF032066.1> | *Merluccius Paradoxus* |
| FAO 47 | *A. pegreffii* | KF032066.1 | <https://www.ncbi.nlm.nih.gov/nuccore/KF032066.1> | *Merluccius Paradoxus* |
| FAO 47 | *A. typica* | JQ912690.1 | <https://www.ncbi.nlm.nih.gov/nuccore/JQ912690.1> | *Merluccius Polli* |
| FAO 47 | *A. berlandi* | JN968646.1 | <https://www.ncbi.nlm.nih.gov/nuccore/JN968646.1> | *Merluccius Polli* |
| FAO 47 | *A. typica* | JQ912690.1 | <https://www.ncbi.nlm.nih.gov/nuccore/JQ912690.1> | *Merluccius Polli* |
| FAO 47 | *A. berlandi* | KC121371.1 | <https://www.ncbi.nlm.nih.gov/nuccore/KC121371.1> | *Merluccius Capensis* |
| FAO 47 | *A. pegreffii* | KF551237.1 | <https://www.ncbi.nlm.nih.gov/nuccore/KF551237.1> | *Merluccius Capensis* |
| FAO 47 | *A. pegreffii* | KF032066.1 | <https://www.ncbi.nlm.nih.gov/nuccore/KF032066.1> | *Merluccius Capensis* |
| FAO 47 | *A. pegreffii* | KF032066.1 | <https://www.ncbi.nlm.nih.gov/nuccore/KF032066.1> | *Merluccius Capensis* |
| FAO 47 | *A. pegreffii* | KF032066.1 | <https://www.ncbi.nlm.nih.gov/nuccore/KF032066.1> | *Merluccius Capensis* |
| FAO 47 | *A. pegreffii* | KF032066.1 | <https://www.ncbi.nlm.nih.gov/nuccore/KF032066.1> | *Merluccius Capensis* |
| FAO 47 | *A. pegreffii* | EU718479.1 | <https://www.ncbi.nlm.nih.gov/nuccore/EU718479.1> | *Trachurus Trachurus* |
| FAO 47 | *A. pegreffii* | KF032066.1 | <https://www.ncbi.nlm.nih.gov/nuccore/KF032066.1> | *Trachurus Trachurus* |
| FAO 47 | *A. pegreffii* | KF032066.1 | <https://www.ncbi.nlm.nih.gov/nuccore/KF032066.1> | *Trachurus Trachurus* |
| FAO 47 | *A. pegreffii* | KF032066.1 | <https://www.ncbi.nlm.nih.gov/nuccore/KF032066.1> | *Trachurus Trachurus* |
| FAO 47 | *A. pegreffii* | KF032066.1 | <https://www.ncbi.nlm.nih.gov/nuccore/KF032066.1> | *Trachurus Trachurus* |
| FAO 47 | *A. pegreffii* | KF032066.1 | <https://www.ncbi.nlm.nih.gov/nuccore/KF032066.1> | *Trachurus Trachurus* |
| FAO 47 | *A. pegreffii* | EU718479.1 | <https://www.ncbi.nlm.nih.gov/nuccore/EU718479.1> | *Trachurus Trachurus* |
| FAO 47 | *A. pegreffii* | KF032066.1 | <https://www.ncbi.nlm.nih.gov/nuccore/KF032066.1> | *Trachurus Trachurus* |
| FAO 47 | *A. pegreffii* | KF032066.1 | <https://www.ncbi.nlm.nih.gov/nuccore/KF032066.1> | *Trachurus Trachurus* |
| FAO 47 | *A. pegreffii* | KF032066.1 | <https://www.ncbi.nlm.nih.gov/nuccore/KF032066.1> | *Trachurus Trachurus* |
| FAO 47 | *A. pegreffii* | KF032066.1 | <https://www.ncbi.nlm.nih.gov/nuccore/KF032066.1> | *Trachurus Trachurus* |
| FAO 47 | *A. pegreffii* | KF032066.1 | <https://www.ncbi.nlm.nih.gov/nuccore/KF032066.1> | *Trachurus Trachurus* |
